# Manganese Supplementation in Deer under Balanced Diet Increases Impact Energy and Contents in Minerals of Antler Bone Tissue

**DOI:** 10.1371/journal.pone.0132738

**Published:** 2015-07-15

**Authors:** Jamil Cappelli, Andrés Garcia, Francisco Ceacero, Santiago Gomez, Salvador Luna, Laureano Gallego, Pablo Gambin, Tomás Landete-Castillejos

**Affiliations:** 1 Animal Science Techniques Applied to Wildlife Management Research Group, IREC Section Albacete (CSIC-UCLM-JCCM), Campus UCLM, Albacete, Spain; 2 Sección de Recursos Cinegéticos y Ganaderos, IDR, Universidad de Castilla-La Mancha, Albacete, Spain; 3 Departamento de Ciencia y Tecnología Agroforestal y Genética, ETSIAM, Universidad de Castilla-La Mancha, Albacete, Spain; 4 Department of Animal Science and Food Processing, Faculty of Tropical AgriSciences, Czech University of Life Sciences. Prague 6-Suchdol, Czech Republic; 5 Departamento de Anatomía Patológica, Universidad de Cadiz, Cadiz, Spain; 6 Departamento de Enfermería y Fisioterapia, Universidad de Cadiz, Cadiz, Spain; CINVESTAV-IPN, MEXICO

## Abstract

Bone ash, collagen, Ca and P composition, are considered the main factors affecting mechanical properties in bones. However, a series of studies in bone and antler have shown that some trace minerals, such as manganese, may play a role whose importance exceeds what may be expected considering their low content. A previous study showed that a reduction in manganese in antlers during a year of late winter frosts led to generalized antler breakage in Spain, which included a reduction of 30% of cortical thickness, 27% reduction in impact energy, and 10% reduction in work to peak force. Starting for this observation, we experimentally studied the effects of manganese supplementation in adults and yearling (yearlings) red deer under a balanced diet. Subjects were 29 deer of different age classes (adult n = 19, yearlings n = 10) that were divided in a manganese injected group (n = 14) and a control group (n = 15). Antler content in ashes and minerals, intrinsic mechanical properties and cross section structure were examined at 4 points along the antler beam. A one way ANOVA (mean per antler) showed that in yearlings, manganese supplementation only increased its content and that of Fe. However, in adults, Mn supplementation increased the mean content per antler of Ca, Na, P, B, Co, Cu, K, Mn, Ni, Se (while Si content was reduced), and impact work but not Young’s modulus of elasticity, bending strength or work to peak force. A GLM series on characteristics in the uppermost part examined in the antler, often showing physiological exhaustion and depletion of body stores, showed also a 16% increase in work to peak force in the antlers of the treated group. Thus, manganese supplementation altered mineral composition of antler and improved structure and some mechanical properties despite animals having a balanced diet.

## Introduction

Antler, which is true bone despite being used when dry and having some unusual features such as the lowest ash content of all bones [1], has attracted the attention of researchers in bone mechanics [2, 3, 4] because, among mammalian bones, it has the highest work to peak force, that is, the amount of work needed to break specimens [2], and it is difficult to break in impact [1, 5]. Furthermore, a series of studies [6–8] have shown that antlers are an excellent model for studying bone biology: i) because they are easily accessible without surgery; ii) because they grow quickly (with an average of 0.67 cm/d [9] in red deer) and demand a high mineral transfer from the skeleton [10]. So that because this fast growth leaves little room for remodeling [11], and thus shows more clearly nutrition or other effects than in internal bone, which is a mosaic of parts build in different times of the life of an animal.

Previous studies by our group have shown that the composition, mechanical properties, and structure of deer antlers (and composition of internal bones too, [12]), or even their histology [11, 13] could be influenced by diet, and natural factors likely affecting mineral composition of plants on which deer feed [4, 6, 7, 14].

Among the minerals that may affect the mechanical properties of antlers, at least one seems to have a disproportionate importance considering its small content in antlers: Mn. In 2010, a study by Landete-Castillejos et al. [4], concluded that among the changes in composition produced in deer antlers by an event of extraordinary low temperatures at the onset of plant sprout, it was Mn decrease which produced a 27% reduction in impact energy, 10% reduction in work to peak force, 30% reduction in antler weight, and 18% reduction in cortical thickness.

It was not the first time that Mn was shown to have an important role in bone. An early study by Strause et al. [15] showed that a balanced diet with low Mn content kept rats in apparently good condition, but their bones had lower Ca content (however, no mechanical tests were performed) and moreover, a study by Leach et al. [16] showed that the addition of Mn in chick cartilage in an ex vivo preparation enhanced significantly the synthesis of main cartilage constituents. However, so far no study has tried to assess the effect of Mn supplementation on both composition, mechanical properties, structure and density of antler or other types of bone.

Thus, we set out to assess the effect of manganese in deer antlers both in adults and in yearlings (which are under a greater growth constraint because they need Ca and other minerals to grow their skeleton in addition to growing their antlers–[9, 17]). The strength and stiffness of whole bones is the result of combination of the overall architecture, such as cortical thickness, and bone material properties [10]. Histology was performed in order to assess if bone mineralization was impaired by Mn supplementation at dosis used (Mn toxicity). Thus, we measured modulus of elasticity *E*, bending strength *BS*, work to peak force *W*, which is the work needed to reach the maximum force [2, 18, 19], and impact energy absorption *U*.

## Materials and Methods

### Study site and antler collection

This study was performed in the Experimental Farm of Universidad de Castilla–La Mancha in Albacete, south-eastern Spain (38°57′10′′N, 1°47′00′′W, 690 m altitude) during 2010. Ours and other research groups regularly perform experiments in these premises (managed by our group) and no specific authorisation is needed to work here, although experiments have to be approved by the Committee of Ethics in Animal Experimentation (Comité de Ética en Experimentación Animal, CEEA) from the Universidad de Castilla-La Mancha (for our experiment, the authorisation number was 1002.04).

Animals were kept in a 10,000 m^2^ open door enclosure on an irrigated mixed pasture. Deer were feed *ad libitum* with a diet of hay, Lucerne, corn and orange pulp; to optimize management time in the experimental farm, all the diet's ingredients were homogeneized and cut in small portions in a tractor-driven commercial mixer.

All animals were adapted to routine management and maintained in good health and body condition during the experiment. Handling procedures and sampling frequency were designed to reduce stress and health risks for the animals [20].

The animals were divided in two groups matched for body measurements such as weight and body condition. Rather than offering Mn mixed with salt, or with the food, which would not allow to control the exact amount of food given to each animal, and also it would mean that control animals had to be placed in another enclosure thus modifying the social environment, we decided to keep all animals together, and deliver the Mn by injections of an aqueous 4% of Manganese gluconate (C_12_H_22_MnO_14_) solution (5cm^3^/100Kg live wt) in the treatment group, given every seven days from start of January to midAugust. The control group was injected a physiological saline solution. Each group counted animals with different ages: for the control group there were 8 adults, 2 subadults (2.5 years old) and 5 yearlings (1.5 years old), for the treatment group there were 8 adults,1 subadult and 5 yearlings. We decided to include subadults to increase group size of the adult class. Thus, the statistical analysis examined yearlings and adults.

Antlers were cut off 1 cm above the burr for safety reasons when they were clean from velvet. We kept carrying out body measurements at the beginning of the trial, during and at the end of the experiment, to monitor potential changes in the animals.

Before the antlers had been removed, they were analyzed according to the standard method for the trophy evaluation in red deer, in order to find a *validation score* for each antler collected [21]. Measurements included: the total length of the main beam, lengths of all the tines, perimeters at three points along the main shaft (burr, between the first tine and the central tine, between the central tine and the crown), total weight of the antlers, and number of tines.

### Sample collection and preparation

Because the deer had a balanced diet much better than deer kept in the wild without supplementation [7, 8, 14], and because the studies performed so far in antler characteristics show a more marked effect of nutrition and other factors during the growing of the antlers main beam [4, 6–8, 11, 13], we analysed, as in other studies [4], effects of Mn supplementation in four positions along the antler beam: *position 1* is directly above the burr, *position 2* referred to the first third of antler shaft, *position 3* after the central tine and *position 4* below the crown (upper tines). For yearlings, antlers were sampled following a similar procedure but including only two sampling points due to the smaller size and different structure of the antlers: one point was close to the base (yearlings have no burr), and the other one 5 cm below the tip [6].

From each position were obtained a 1 cm-slice (complete transverse cross-section) and a cylinder of about 5–6 cm (to obtain bars from the cortical wall).

Bars were identified according to individual deer and level in the main beam. The outer or periosteal side was also marked in order to test always with this side in tension, and to mark the end closer to the antler base.

A circular low-speed saw was used for the initial cutting of the antler. Then, the surfaces were abraded using a semiautomatic equipment for polishing (Struers LaboPol-21, Denmark) to get the right size of bars. Care was taken to produce parallel surfaces, and the width and depth of each sample were recorded to the nearest 0.01 mm using a digital calliper prior to testing. The final size was 4.5 mm wide, 2.5 mm deep, and a variable length but always allowing a gauge length of 40 mm, a length in which shear effects reducing calculated *E* are very small [4].

In addition, antlers of adult groups were examined by histology. Antlers were sampled in position 1 and 4 were embedded in polymethyl-methacrylate. Mineralized sections (50 μm-thick) were prepared by grinding-polishing method, and sections were stained with toluidine blue/pyronin G for light microscopy examination [13]. In order to increase the contrast between the trabecular and cortical bone to better measure the cortical thickness and derived variables, antler slices were coloured with a solution of ink/water in proportion of 1:4 (Pelikan ink 4001, Germany) and dried for 24 hours at room temperature. Then, the slices were polished to increase the contrast between cortical and trabecular bone. Once the cortical part had no remains of ink, each slice was scanned on a flatbed scanner (Ricoh aficio MP C2800) at 600 dpi and measured in an image analysis software (ImageJ), where cortical bone thickness of the cross-sections was measured at six equally spaced points around the perimeter. After this, we calculated the average thickness as the mean of these 6 measurement points. In addition, we measured the total area of the section, the area of cortical and trabecular bone. These areas were used to calculate, in turn, the ratio between cortical and total area (termed *ratio cortical/total*) [4].

### Bone mechanical properties

Specimens for mechanical testing were first subjected to a procedure to standardize humidity content. For this, they were first introduced in a buffer solution (BioWhittaker HBSS: Hank's balanced salt solution, Belgium) for 48 hours. This is a way to achieve maximum water content, but at the same time minimizing the risk that small amounts of mineral may leach out of the bone [1]. After this, the bars were dried in the laboratory, at a temperature of approximately 20°C and relative humidity of 40% for a period of 72 hours. The bars that had thus homogeneous humidity content were tested in three-point bending with the periosteal side in tension. This was carried out in a Zwick/Roell 500N machine. The speed of the machine head was set to 32 mm/min. Gauge length or distance between supports was set to 40 mm. Machine compliance was tested and found to be negligible at this gauge length and sample depth.

The mechanical properties measured were Young's Modulus of elasticity, an estimate of stiffness; Bending strength, calculated from the maximum stress at the greatest load borne following the procedure for this and the other intrinsic mechanical properties [1, 2, 4, 8] and the total work under the load-deformation curve up to the maximum load borne, divided by cross-sectional area (Work to peak force). Total work normalized like this gives some idea of the toughness of the specimen. Although the software offers the mentioned mechanical properties, the key parameters for the formula used were extracted from the output chart in the software testXpert II (Zwick GmbH & Co, Ulm, Germany) and calculated in Excel following the formulae indicated, as our own studies have shown that there are differences between results calculated and derived from the machine, depending on gauge length, toe region excluded from the start of the curve in calculus of *E*, and others.

The impact testing procedure consists, essentially, of a pendulum falling on, and breaking an un-notched sample with the periosteal side in tension. The loss of kinetic energy of the pendulum is measured by the machine, and this is considered to be the energy required to break the sample [4]. This energy is normalized by dividing by the cross-sectional area of the specimen, producing the impact energy absorption or impact work. Tests were carried out in a CEAST-IMPACTOR II testing machine (CEAST S.p.A., Pianezza, Italy) with manual releasing device. A hammer with a potential energy of 1J was used for all tests.

### Chemical analysis

Breaking one antler bar in impact and one in bending test produced 4 similar-size fragments. One of these fragments was used to assess the content of different minerals. For this, the side of the fracture was polished to have a regular shape and also in areas where it may have any pen mark. Then, in order to calculate the specific density, the specimen was placed in a controlled heating chamber for 72 hours at 60°C to dry it out fully, and afterwards each sample was weighed with a precision balance (±0.01 g) and measured with a precision scale (±0.01 mm). Density was calculated dividing the weight by the volume (calculated as length x width x depth). After this, the segment of bar was placed in a tube for mineral analysis (details on the method used for mineral analysis are described in [4]).

Another fragment was used to measure ash content. For this, the specimen was heated for 72 hours at 60°C. Then, the samples were weighed with a precision balance (±0.01 g) to get the dry weight, and subsequently was placed in a muffle furnace (HTC 1400, Carbolite, UK) for 6 hours at 480°C. Ash content was calculated as the value of ashes thus obtained divided by the dry weight.

### Statistical analysis

One-way ANOVAs were used to check for differences in body parameters between groups before being subjected to manganese treatment. The same statistical test was used to assess rough differences in body weight after the experiment by Mn treatment, also and in each parameter assessed in whole antler. We also examined the remaining antler variables by performing the ANOVA on the mean of the 4 antler levels. The antler variables tested were: cortical thickness; ratio cortical to total antler section area of the bone slice, in proportion; antler length; antler score as trophy; specific density/gravity of the cortical bone; Young’s modulus of elasticity *E*; bending strength *B*; work to peak force *W*; impact energy *U*; ash content and that of minerals Ca, P, Mg, Na, K, B, Co, Cu, Fe, Mn, Ni, S, Se, Si, Sr, and Zn. Because data showed a very clear difference between yearlings and adults, we performed a GLM, testing the effects of treatment and age class on each of the studied antler variables, and after results confirmed this pattern, the following statistical tests were carried out separately for yearlings and adults. The second part of the analyses involved a series of general linear models (GLMs) to assess the treatment effects on the same variables once the effects of body weight (which greatly influence antler size and characteristics) was included in the models. Three different sets of analyses examined: 1) the average value for the four sampled positions in the antler; 2) the proportional difference between the top and the base (*Position 4*—*Position 1* / *Position 1*; *i*.*e*. a decrease in values is shown as a negative difference in percent); 3) finally, the values at the fourth level sampled in the antler. GLMs 2 and 3 were carried out because values often show a variation between base (which shows the condition of the animal at the start of antler growth), and top part of the antler (when different value in most parameters shows the physiological effort made to grow the antler: [6–8]).

All analyses were carried out with SPSS version 19 (SPSS Inc., Chicago, IL, USA).

### Ethical statement

This study was carried out in strict accordance with the Spanish legislation for the use of animals in research (law 6/2013 and 32/2007). The protocol was approved by the Committee of Ethics in Animal Experimentation (Comité de Ética en Experimentación Animal) from the Universidad de Castilla-La Mancha (Permit Number: 1002.04). Because antlers are dead when they are hard and clean of velvet [1], antler removal produces no pain and no anesthesia is needed. Nevertheless, a low dose of xylazine (0.3 mg/kg body mass) was used as tranquilizer to reduce stress and minimize suffering. Animals were restrained using a cushioned crush specifically designed to restrain deer movements, then the tranquilizer was delivered in an intravenous injection and, after one minute, antlers were cut using a low speed electrical saw. In general, this and all other experimental handling was designed to minimize stress and suffering of deer.

## Results

**Fig 1:**
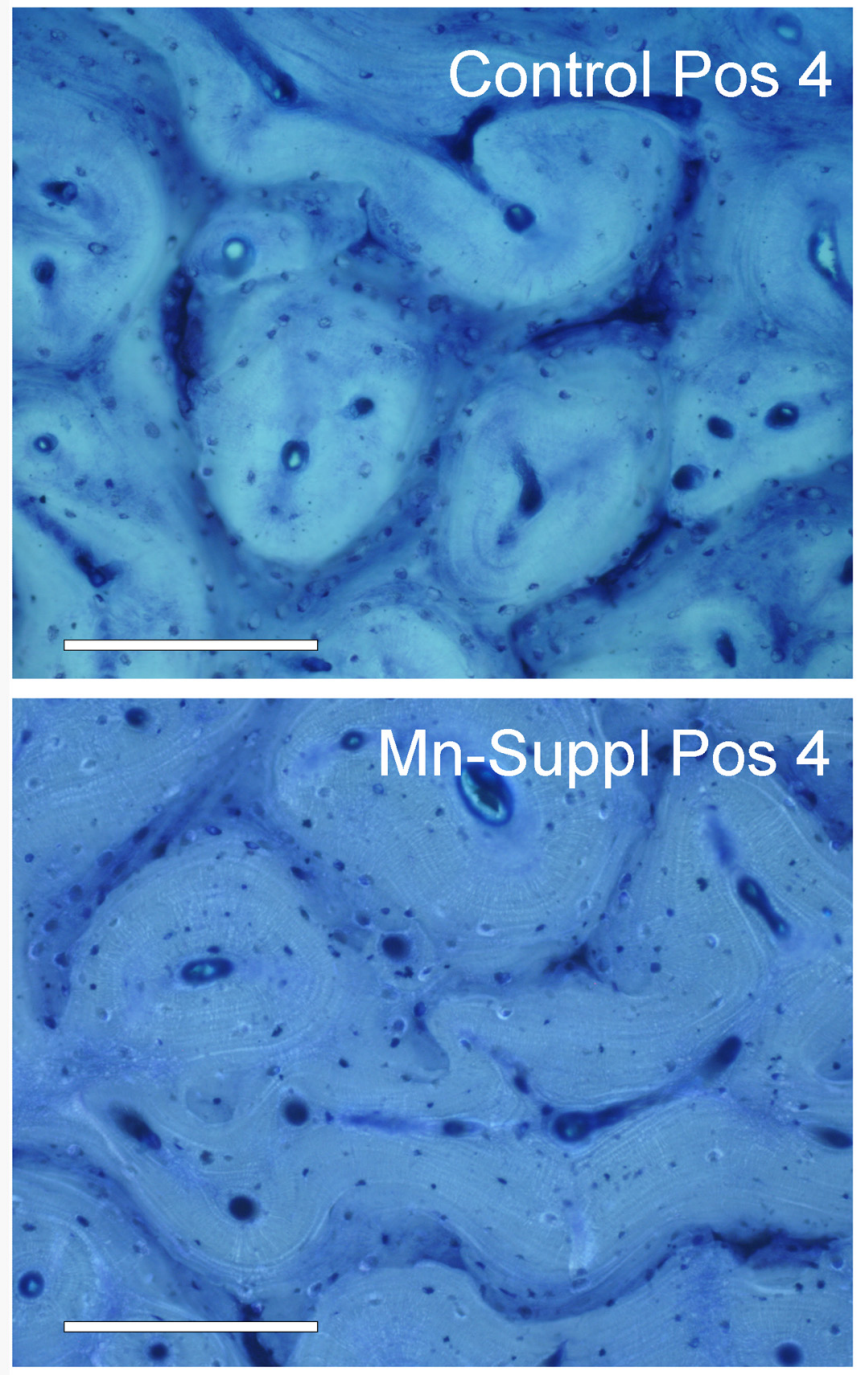
Histology of cortical bone in antlers from control and Mn-supplemented adult groups. Note that primary osteons are complete in both cases. Mn-supplemented osteons looks normal, osteomalacic seams—an indicator of Mn toxicity—are not observed (Bar scale = 200 μm).

Throughout the analyses, results from yearlings showed a very different pattern to that of adults. A set of GLMs including treatment and age class showed that they differ in all mechanical properties (greater values for adults), all antler structure and measurements (except average cortical thickness), and also in B, Cu, Fe, Mn, Ni, S, Si and Zn. Histology did not reveal any alteration in Mn-supplemented antlers. Primary osteons were completely formed both at position 1 and 4 (**Fig 1**), osteons in cortical bone did not show osteomalacic seams (which is considered a sign of Mn toxicity).

Results are not shown for sake of brevity, more supporting information in **S1 Dataset** (although means can be assessed in Table 1). When both age classes were tested separately in one-way ANOVAs, the only treatment effect which showed a common statistically significant effect was the increase in manganese content in the antler of the treatment group (Table 1). Manganese in antlers of animals injected was 2.5 times greater for yearlings (compared to the control group), and 2.3 times for antlers of adults. Yearlings only showed a 3-fold increase in antler content of Fe, but no other effect. As any other effect was found in any GLM in the group of yearlings, the rest of the analyses shown regard only to the adults group, for concision purposes.

**Table 1:** Antler characteristics of spiker and adult red deer injected (Mn treated) or not (Control) with manganese as nutrient supplement. P corresponds to one-way ANOVA on the mean (± SE) per antler of the four positions examined.

	Yearlings			Adults		
Variables	Mn-Treatment	Control	P	Mn-Treatment	Control	p
Young’s modulus of elasticity (*E*), Gpa	12.0 ± 2.2	11.3 ± 1.8	Ns	14.4 ± 0.4	13.9 ± 0.8	ns
Bending Strength (*BS*), Mpa	232.8 ± 42.0	202.3 ± 35.2	Ns	267.9 ± 6.1	266.7 ± 15.4	ns
Work to peak force (*W*), kJm^-2^	35.2 ± 7.1	27.3 ± 6.8	Ns	44.0 ± 1.0	42.9 ± 1.3	ns
Impact work (*U*), kJm^-2^	14.9 ± 2.2	13.7 ± 1.5	Ns	17.7 ± 0.6	15.8 ± 0.6	0.050
Body weight difference (%)	54.4 ± 2.7	61.1 ± 4.2	Ns	19.8 ± 1.7	30.9 ± 2.5	0.003
Cortical/total Ratio	0.8 ± 0.03	0.7 ± 0.1	Ns	0.5 ± 0.01	0.5 ± 0.03	ns
Average cortical thickness (mm)	5.7 ± 0.6	5.1 ± 0.7	Ns	5.6 ± 0.3	6.2 ± 0.4	ns
Antler length (cm)	52.0 ± 5.0	48.9 ± 3.7	Ns	83.2 ± 4.1	88.3 ± 3.7	ns
Antler score	56.8 ± 4.0	60.0 ± 1.8	Ns	147.1 ± 8.0	154.6 ± 7.2	ns
Specific gravity of cortical bone (g/mL)	1.6 ± 0.18	1.4 ± 0.2	Ns	1.8 ± 0.1	1.7 ± 0.5	ns
Ashes (%)	60.0 ± 6.8	52.8 ± 7.9	Ns	58.7 ± 5.7	51.0 ± 2.7	ns
Ca (wt%)	19.7 ± 2.2	17.4 ±2.6	Ns	21.9 ± 0.1	20.2 ± 0.5	0.013
Mg (wt%)	0.4 ± 0.5	0.4 ± 0.5	Ns	0.5 ± 0.01	0.5 ± 0.01	ns
Na (wt%)	0.5 ± 0.1	0.50 ± 0.1	Ns	0.6 ± 0.01	0.53 ± 0.01	<0.001
P (wt%)	9.5 ± 1.1	8.5 ± 1.3	Ns	10.4 ± 0.03	9.5 ± 0.2	0.002
B (ppm)	4.6 ± 0.6	4.0 ± 0.6	Ns	6.0 ± 0.1	4.2 ± 0.2	<0.001
Co (ppm)	0.5 ± 0.0	0.4 ± 0.1	Ns	0.6 ± 0.01	0.3 ± 0.05	<0.001
Cu (ppm)	0.7 ± 0.1	0.6 ± 0.1	Ns	1.0 ± 0.04	0.8 ± 0.03	<0.001
Fe (ppm)	12,0 ± 2.7	4.3 ± 1.2	0.045	3.8 ± 1.9	3.4 ± 0.9	ns
K (ppm)	655.6 ± 80.3	573.4 ± 113.3	Ns	635.6 ± 17.2	431.9 ± 38.8	<0.001
Mn (ppm)	0.5 ± 0.1	0.2 ± 0.04	0.003	0.7 ± 0.4	0.3 ± 0.2	<0.001
Ni (ppm)	0.4 ± 0.1	0.3 ± 0.06	Ns	0.7 ± 0.5	0.4 ± 0.6	<0.001
S (ppm)	1045.2 ± 126.9	935.5 ± 150.1	Ns	1177.3 ± 15.1	1126.1 ± 24.3	ns
Se (ppm)	3.1 ± 0.4	2.9 ± 0.7	Ns	4.1 ± 0.2	1.7 ± 0.5	<0.001
Si (ppm)	27.4 ± 3.9	20.9 ± 4.1	Ns	32.7 ± 2.0	59.6 ± 11.5	0.044
Sr (ppm)	1003.9 ± 103.1	725.2 ± 101.8	Ns	784.5 ± 54.3	754.3 ± 31.9	ns
Zn (ppm)	63.6 ± 7.9	57.9 ± 7.5	Ns	84.3 ± 4.0	77.8 ± 4.8	ns

A first exam of results regarding Mn supplementation in adults group, showed that body weight increased by 10% less in the supplemented group from start to end of the experiment than in the control group (Table 1). In contrast, Ca and P increased 8% and 10% respectively. Most macrominerals and trace elements also increased. Thus Na increased 14%, K increased 47%, Se increased 142%, and Cu increased 29%. B, Co, and Ni also increased, whereas Si was the only mineral that decreased. No other minerals showed significant differences until effect of body weight were included in the GLMs. Among structural and mechanical properties Mn supplementation produced 11.8% increase in impact energy, but no other mechanical or structural property was affected. There was also an increase in impact energy in yearlings lower than that in adults (9%), but this was non-significant because of the large variability within each group.

A more detailed set of analyses, controlling for the effect of weight, showed more clearly the effects of Mn in the antlers of adults, particularly in the upper sections of the antler where physiological exhaustion is more visible (Tables 2–4). Table 2 shows the effects of Mn supplementation in the mean values of the four sections of the antlers which were tested. Controlling for body weight did not reveal any further effect as compared to the ANOVA, except that Co, K, Mn, Se and Si were both affected by Mn supplementation and deer body weight. In addition, body weight but not Mn supplementation, affected average values in cortical thickness, antlers length, specific density/gravity, Young’s modulus of elasticity, Bending strength, and Zn content. In the rest of the antler parameters, Mn treatment had only an effect on impact energy (*U*: Table 2), but in no other mechanical variable nor structural one.

**Table 2:** General Linear Model analyses showing the influence of treatment (injections of Manganese) and weight, on the composition, structure and mechanical properties of antlers in adult red deer. The coefficient β (± S.E.) is related to the difference of the value observed in animals that were injected with respect to the group not injected. Dashes indicate coefficients that were not significant.

			Factors in the model			
			Mn-Treatment		Weight (Kg)	
Variables	R2	Intercept ± S.E.	β ± S.E.	Sig.	Β ± S.E.	Sig.
Young’s modulus of elasticity (*E*), Gpa	0.50	6.8 ± 1.8	-	-	-0.04 ± 0.01	0.001
Bending Strength (*BS*), Mpa	0.51	128.8 ± 33.5	-	-	-0.7 ± 0.2	0.001
Work to peak force (*W*), kJm^-2^	-	-	-	-	-	-
Impact Work (*U*), kJm^-2^	0.20	17.7 ± 0.6	1.9 ± 0.9	0.05	-	-
Cortical/total ratio	-	-	-	-	-	-
Average cortical thickness (mm)	0.67	1.16 ± 0.8	-	-	-0.02 ± 0.004	<0.001
Antler length (cm)	0.55	39.0 ± 10.5	-	-	-0.2 ± 0.05	<0.001
Antler valuation score	0.61	55.6 ± 18.9	-	-	-0.5 ± 0.1	<0.001
Specific gravity of cortical bone (g/mL)	0.34	1.4 ± 0.1	-	-	-0.002 ± 0.001	0.009
Ashes (%)	-	-	-	-	-	-
Ca (wt%)	0.31	21.9 ± 0.4	1.6 ± 0.6	0.013	-	-
Mg (wt%)	-	-	-	-	-	-
Na (wt%)	0.62	0.6 ± 0,01	0.07 ± 0.01	<0.001	-	-
P (wt%)	0.44	10.4 ± 0.2	0.9 ± 0.3	0.002	-	-
B (ppm)	0.73	6.0 ± 0.2	1.7 ± 0.2	<0.001	-	-
Co (ppm)	0.71	0.9 ± 0.1	0.3 ± 0.05	<0.001	0.002 ± 0.001	0.034
Cu (ppm)	0.59	1.0 ± 0.03	0.2 ± 0.05	<0.001	-	-
Fe (ppm)	-	-	-	-	-	-
K (ppm)	0.75	961.0 ± 94.7	194.0 ± 34.1	<0.001	1.7 ± 0.5	0.003
Mn (ppm)	0.85	0.4 ± 0.1	0.4 ± 0.04	<0.001	-0.001 ± 0.001	0.045
Ni (ppm)	0.57	0.7 ± 0.1	0.4 ± 0.1	<0.001	-	-
S (ppm)	-	-	-	-	-	-
Se (ppm)	0.67	7.4 ± 1.3	2.3 ± 0.5	<0.001	0.02 ± 0,01	0.019
Si (ppm)	0.46	-43.7 ± 29.4	-24.6 ± 10.6	0.034	-0.4 ± 0.1	0.016
Sr (ppm)	-	-	-	-	-	-
Zn (ppm)	0.29	41.6 ± 15.2	-	-	-0.2 ± 0.1	0.018

**Table 3:** Mean ± SE differences between the basal position (*Position 1*) and the distal position (*Position 4*) in percentage, in adult red deer injected (Mn-Treatment) or not (Control) with manganese as a nutrient supplement. A decrease from base to top is shown as a negative value. The right half of the table shows the mean ± SE for position 4 (the uppermost showing most clearly physiological exhaustion and depletion of body stores). Differences and probabilities are shown on the basis of one-way ANOVAs.

	Difference base/tip (%)			Position 4		
Variables	Mn-Treatment	Control	P	Mn-Treatment	Control	P
Young’s modulus of elasticity (E), Gpa	-6.8 ± 0.4	-9.4 ± 4.0	Ns	13.9 ±0.9	13.2 ± 1.0	ns
Bending Strength (BS), Mpa	-5.2 ± 3.2	-5.7 ± 3.4	Ns	262.9 ±12.3	257.3 ± 17.6	ns
Work to peak force (W), kJm^-2^	6.6 ± 7.7	-1.9 ± 6.6	Ns	46.9 ±1.9	42.3 ±2.9	ns
Impact work (U), kJm^-2^	-12.9 ± 6.2	-8.5 ± 54.7	Ns	15.0 ± 0.8	15.2 ± 1.1	ns
Cortical/total ratio (%)	-32.8 ± 5.8	-28.7 ± 5.1	Ns	0.4 ± 0.02	0.4 ± 0.03	ns
Average cortical thickness (mm)	-27.2 ± 5.4	-19.9 ± 4.9	Ns	4.7 ± 0.4	5.4 ± 0.3	ns
Specific gravity of cortical bone (g/mL)	-2.0 ± 0.7	-3.8 ± 1.4	Ns	1.7± 0.02	1.7 ± 0.3	ns
Ashes (%)	-0.6 ± 0.3	-1.4 ± 1.6	Ns	63.9 ± 0.3	62.6 ± 0.6	ns
Ca (wt%)	-1.3 ± 1.6	-1.6 ±1.2	Ns	21.6 ± 0.2	20.5 ± 0.1	<0.001
Mg (wt%)	1.8 ± 3.2	-3.9 ± 1.1	Ns	0.5 ± 0.02	0.5 ± 0.01	ns
Na (wt%)	-1.6 ± 3.1	-5.9 ± 1.7	Ns	0.6 ± 0.01	0.5 ± 0.01	0.002
P (wt%)	-1.6 ± 0.8	-1.1 ± 0.8	Ns	10.3 ± 0.1	9.7 ± 0.1	<0.001
B (ppm)	6.5 ± 3.9	-0.2 ± 4.9	Ns	6.1 ± 0.2	4.5 ± 0.3	<0.001
Co (ppm)	9.8 ± 0.5	13.1 ± 7.1	Ns	0.6 ±0.02	0.3 ± 0.1	<0.001
Cu (ppm)	15.4 ± 7.9	5.7 ± 8.8	Ns	1.1 ± 0.1	0.9 ± 0.7	ns
Fe (ppm)	92.8 ± 72.9	83.3 ± 35.3	Ns	2.8 ± 1.6	7.5 ± 2.7	ns
K (ppm)	9.7 ± 3.6	0.4 ± 3.2	Ns	669.3 ± 22.7	472.8 ± 56.8	0.005
Mn (ppm)	-0.6 ± 4.3	-10.0 ± 2.6	0.057	0.7 ± 0.05	0.4 ± 0.1	0.006
Ni (ppm)	21.8 ± 12.7	5.2 ± 11.9	Ns	0.8 ± 0.1	0.5 ± 0.1	ns
S (ppm)	7.9 ± 2.9	4.9 ± 3.0	Ns	1237.9 ± 19.1	1185.9 ± 37.6	ns
Se (ppm)	10.1 ± 8.3	15.4 ± 21.4	Ns	4.2 ± 0.3	2.3 ± 0.8	0.030
Si (ppm)	6.1 ± 20.3	52.4 ± 55.7	Ns	34.4 ± 3.8	58.6 ± 12.7	ns
Sr (ppm)	4.5 ± 1.2	3.3 ± 1.2	Ns	799.2 ± 51.9	760.8 ± 31.5	ns
Zn (ppm)	3.7 ± 2.3	1.8 ± 1.8	Ns	85.9 ± 4.0	83.6 ± 4.0	ns

**Table 4:** Influence of injections of Mn (as nutrient supplement) and body weight, on the composition, structure and mechanical properties of antlers in the distal position (position 4), in adult red deer; the factor β is related to the difference of the value observed in animals that were injected with respect to the group not injected. Using GLMs analysis for each variable. Dashes indicate coefficients that were not significant.

			Factors in the model			
			Mn-Treatment		Weight (Kg)	
Variables	R2	Intercept ± S.E.	β ± S.E.	Sig.	Β ± S.E.	Sig.
Young’s modulus of elasticity(*E*),Gpa	0.50	6.8 ± 1.8	-	-	-0.04 ± 0.01	0.001
Bending Strength (*BS*),Mpa	0.50	128.8 ± 33.5	-	-	-0.7 ± 0.2	0.001
Work to peak force (*W*),kJm^-2^	0.41	26.1 ± 8.3	7.1 ± 3.1	0.036	-0.1 ± 0.04	0.020
Impact work (*U*),kJm^-2^	-	-	-	-	-	-
Cortical/total ratio(%)	-	-	-	-	-	-
Average cortical thickness(mm)	0.47	0.9 ± 1.1	-	-	-0.02 ± 0.005	0.001
Specific gravity of cortical bone(g/mL)	0.42	1.4 ± 0.1	-	-	-0.001 ± 0.0004	0.004
Ashes (%)	-	-	-	-	-	-
Ca (wt%)	0.55	21.6 ± 0.2	1.1 ± 0.3	<0.001	-	-
Mg (wt%)	-	-	-	-	-	-
Na (wt%)	0.47	0.6 ± 0.01	0.1 ± 0.02	0.002	-	-
P (wt%)	0.60	10.3 ± 0.1	0.6 ± 0.1	<0.001	-	-
B (ppm)	0.54	6.1 ± 0.3	1.7 ± 0.4	<0.001	-	-
Co (ppm)	0.56	0.6 ± 0.04	0.3 ± 0.1	<0.001	-	-
Cu (ppm)	-	-	-	-	-	-
Fe (ppm)	-	-	-	-	-	-
K (ppm)	0.39	669.3 ± 43.2	196.5 ± 61.2	0.005	-	-
Mn (ppm)	0.38	0.7 ± 0.1	0.3 ± 0.1	0.006	-	-
Ni (ppm)	-	-	-	-	-	-
S (ppm)	-	-	-	-	-	-
Se (ppm)	0.29	4.2 ± 0.5	1.9 ± 0.8	0.030	-	-
Si (ppm)	-	-	-	-	-	-
Sr (ppm)	-	-	-	-	-	-
Zn (ppm)	-	-	-	-	-	-

A set of GLMs on the difference between tip and base of the antler (expressed as percentage), which shows the depletion of body mineral stores or physiological exhaustion, showed no effect of treatment except on Mn levels. The former did not decrease in the supplemented group as clearly as they decreased in the control group (the mean comparison of both groups is shown in Table 3, using ANOVAs).

Some further effects were revealed in analyses regarding the distal position. Table 3 shows the means for the uppermost position examined in the antler (just below the crown). Although most minerals (except Cu) showed a similar effect than the analysis conducted on the mean antler content, ash content in the treated group was 13 percent units higher (Table 3) than in the control group. One of the most interesting differences regarded the effect on work to peak force, once the effect of body weight was controlled for. Thus, supplementation with Mn increased work to peak force (β for untreated group = -7.1 ±3.1; p = 0.036; shown in Table 4).

## Discussion

Results show clearly that manganese supplementation is reflected in antlers of both yearlings and adults. It is particularly interesting the double pattern found in the influence of manganese supplementation in mineral composition: whereas antlers of yearlings were not affected in any mineral except iron, those of adults, despite deer having the same balanced diet, Mn-treatment increased content in Ca, P, Na, K, Se, B, Co, Ni and Cu. As expected, Mn had also a positive effect increasing the impact energy and also the work to peak force, but in this latter case only after body weight effect was controlled for in GLM analysis. No other effects in mechanical properties nor structural variables were found.

The fact that manganese supplementation was clearly reflected in antlers in both yearlings and adults follows matches previous results showing that antlers reflect the composition of the diet [4, 7, 14], as it happens also with trace elements in internal bones, at least for the case of deer [12]. A particular difference in our case is that manganese was not ingested orally but injected. This probably means that manganese raised its content in blood in the animals treated, although we have no measure on this.

The study gives support to findings of a previous study which propose a change in Mn content of plants as a potential cause of breakage of many antlers in Spain: an effect of climate reduced work to peak force in antlers by 10%, impact energy by 27%, cortical thickness by 30%, and also affected content in ash, Ca, P, Mn, Si, Na, Co, Cu (in this case only with marginal significance), and Fe. Two characteristics are particularly interesting in this comparison: 1) both studies show nearly the same effects in antler composition, despite the difference between a manganese supplementation in deer under a balanced diet, and a climatic event affecting plant composition in the wild; 2) manganese affected most minerals in our farm study despite the animals being under a rich and balanced diet (which is only low in manganese, as in most other studies with deer fed wholemeal under a farming setup [4, 22]). However, with the doses used no signs of Manganese toxicity were appreciated by histology. So the amount of Mn injected was safe. “Manganese rickets” (a disorder reported with excessive amounts of Mn in the diet–[23]) was not observed. In the study mentioned above [4], it was hypothesized that the stress caused in plants by a period of extraordinary frosts produced an increase in Si (a generalized response of plants to stress: [24–26]), and this, in turn, produced the reduction in Mn, Na, and probably other minerals; then the effect of Mn possibly influenced reduction in Ca and P. Surprisingly, in the present study, even under a balanced diet, a supplementation with Mn increased ash content, that of Ca, P, Na, Co, Se and B, and reduced the content of Si, which are exactly the same effects shown in the 2010 study (except that Fe decreased with Mn induced deficiency by the climatic effect in that study, but there is no effect here, and K was not affected in that study but it is in the present one). As indicated in the second point, it is remarkable that such effects have been found in our study in animals under a balanced diet. This would explain why structural properties were not affected in the present case, as the control group is not feeding on plants growing in a dry year with exceptional frosts [4], but feeding on a rich protein, balanced mineral diet, as was the case in the treatment group.

Before discussing the effect of Mn in adults, we would like to bring the attention of the reader to the fact that no effect is found in other minerals or mechanical properties of yearlings. The reason may be that these animals are under a strong constraint for growth, so that influences in antler characteristic are smaller than in adults. There are two lines of evidence supporting this hypothesis: 1^st^) most antler characteristics are different between yearlings and adults (except cortical thickness, all structural and mechanical variables, plus concentration of B, Cu, Fe, Mn, Ni, S, Si and Zn). 2^nd^) the increase produced by Mn injection as compared to the control is smaller in yearlings than in adults in mineral composition: Na (+13.7% in adults, +11.5% in yearlings), B (+40.9% adults,+14.6% yearlings), Co (+99.6% in adults,+12% yearlings), Cu (+29.5% adults, +25.2% yearlings), K (+47% adults, +14% in yearlings), Ni (+108% in adults,+26% in yearlings) and Se (+142.7% in adults, +7.5% in yearlings). The trend is not so clear in other antler characteristics.

Why does Mn affect impact energy and mineral composition? Whereas it is not so clear why supplementation affects mineral composition of so many minerals, we have a relatively abundant literature that is suggestive of why Mn is particularly important in impact energy and work to peak force. It was shown long ago that Mn is found mainly in the mineral, and a small proportion is found in the organic matrix [27]. Thus, because Mn2+ can substitute Ca2+ in the apatite lattice [28], at first glance one may think that the effect of Mn supplementation may be caused by its storing in the skeleton. However, despite the fact that 25% of Mn in human body is stored in this way [29], it is a coenzyme for glycosyltransferases, which create glycosaminoglycans (GAGs), and sulfonases, which sulfate these molecules in the final step to produce proteoglycans [29, 30]. One of the main GAGs is chondroitin sulphate, the major constituent of cartilage, so that it is long known that Mn deficiency reduces cartilage growth by impairing chondroitin sulphate and other GAG biosynthesis [16, 30] In addition to the study by our group in 2010 [4], we do not know of other studies showing how this affects bone mechanical properties, but in arteries, Mn deficiency has been shown to affect mechanical properties by affecting GAG biosynthesis and sulfation [29, 31]. Thus, it is not surprising that the most important mechanical property influenced by Mn supplementation has been impact energy and, to a lesser extent, work to peak force. GAGs are highly polar and attract water, so that they affect particularly elastic properties and this is the reason why they are particularly important in arteries and why Mn affects cardiovascular disease in humans [29]. However, the closest evidence to the effect of Mn in a mechanical property similar to impact energy is not found within humans or mammals, but in birds. In addition to the effect of Mn in chick cartilage, recent research has shown that Mn supplementation increases fracture toughness in eggs of laying hens [32]. Fracture toughness is similar to impact energy or work to peak force in antlers in that it measures the energy required to grow a thin crack in an egg shell. The study shows that the well known effect of Mn supplementation in enhancing mechanical properties in eggshell is achieved through increasing the glycosaminoglycan synthesis. However, the situations are completely different and so may be the mechanisms by which in both cases energy required to grow a crack is increased by supplementation of Mn. In fact, for the case of eggs, the effect seems to be achieved by influencing the GAG contents in the eggshell membrane, rather than in the shell itself.

Why Mn increases the content of most minerals in antler? A very likely mechanism is, again, the increase in glycosaminoglycans. Cartilage, which precedes bone and antler mineralization [11], is formed by an extracellular matrix consisting mostly of collagen II and proteoglycan, the latter attached chondroitin sulphate and other GAGs [33]. The author found that, because GAGs have a high fixed negative charge density, GAGs increased in content in cartilage, so did the content in Na^+^. Thus, it is very likely that as Mn increases chondroitin sulphate and GAGs in antlers of adult deer, the increased negative charge may have increased the content of all positive cations (most of these increased, although some, like Mg, Zn, Fe and Sr did not increased as a result of high variability, the rest showing a significantly higher content in antlers of deer injected with Mn, except Si). How could this be possible considering that antler is made of bone rather than cartilage? According to several studies [11, 34, 35] antler is formed first as a scaffolding of cartilage leaving longitudinal tubes that at the end of the process are filled by osteons. Before the creation of osteons, the scaffolding of cartilage is first calcified and subsequently substituted by a bone. In bone, approximately 0,25% is made by non collagenous proteins [36]. One may think that such small remaining amount of GAGs and other non-collagenous proteins are unlikely to create the same effect as found in cartilage [33]. However, on the one hand the greater amount of cations brought by increased GAGs of antler may remain as increased contents in the bone scaffolding, and also they may influence the overall process in which antler bone is formed, and also in the way that cracks grow [37, 38]. Thus, it is particularly interesting that in the histological study, the staining of proteoglycans showed a different pattern between Mn-treated and control antlers: the treated having a more homogeneous distribution of proteoglycans.

In conclusion, the present study is the first one to show that organic Mn injected in deer that otherwise had a balanced diet, affected content of most minerals and also improved mechanical properties related with growth of fractures. In addition, Mn supplementation seemed to influence the distribution of GAGs assessed through histological staining. Thus, the present study may be a first step towards understanding effects in bone of supplementation with Mn in enhancing bone quality and some bone mechanical properties in situations where individuals are not deficient in them.

## Supporting Information

S1 Dataset Table of data collected for deers included in the experiment. Data obtained from the analysis of the chemical composition, the mechanical tests and the study of the structure, using bone samples extracted from antlers of red deer.
(XLSX)
